# Editorial: Engineered Adjuvants and Carriers for an Animal Infectious Disease Vaccine

**DOI:** 10.3389/fbioe.2021.692634

**Published:** 2021-05-21

**Authors:** Sankar Renu, Heather L. Wilson, Kaissar Tabynov, Jagadish Hiremath

**Affiliations:** ^1^The Ohio State University, Wooster, OH, United States; ^2^Vaccine & Infectious Disease Organization, University of Saskatchewan, Saskatoon, SK, Canada; ^3^Kazakh National Agrarian University, Almaty, Kazakhstan; ^4^ICAR-National Institute of Veterinary Epidemiology and Disease Informatics (NIVEDI), Bengaluru, India

**Keywords:** nanoparticles, vaccines, adjuvants, Hsp90, polyphosphazenes, immunity, animal infectious disease

The prevalence of infectious diseases in livestock is not only an economic threat, but also significantly impacts animal and human health and welfare. From ancient times until today, most of the existing and newly emerging infectious diseases have arisen from animal origin and many of them have been zoonotic in nature. Vaccination in animals is the commonly used approach to protect their health as well as to prevent infectious diseases spillover to humans. However, we don't have effective vaccines for many infectious diseases. To improve vaccine efficacy, we seek to formulate them to include immunogenic adjuvants and/or carriers and to administer them via mucosal routes where in most pathogens gain entry into the body. Hence, we have launched this special issue to cite the recent progress of discovery research in novel adjuvant and carriers to improve the animal infectious disease vaccines.

The application of nanoparticles on human diseases is well-studied, but its use is in only the preliminary stage in animal infectious diseases research. In the recent era, chitosan nanoparticles have been used to deliver the animal infectious disease vaccines via different routes including targeting the mucosa by capitalizing on its natural mucoadhesive and adjuvant properties. In this Research Topic Renu and Gourapura, have summarized studies that used chitosan nanoparticles to deliver the bacterial and viral animal infectious disease vaccines through the mucosal routes. Interestingly this review article described how the chitosan nanoparticle-based infectious disease vaccines induced a type of immunity, its mechanism of action in the infections native host of poultry and pigs, and strategies to improve the vaccine efficacy. In another review article Maina et al. highlighted nanovaccine application in the prevention of cattle infectious diseases. This review emphasized recent advancements in synthetic and natural polymeric nanovaccine development and the mechanism by which nanovaccines interact with the bovine immune system. They also deliberated the positive implications of nanovaccines use for combating several important viral and bacterial disease syndromes in beef and dairy cattle.

*Rhipicephalus microplus* (cattle tick) is known to have a high impact on cattle health and the livestock industry in tropical and subtropical regions. Mody et al. used silica vesicles as self-adjuvanting antigen carrier to deliver *Rhipicephalus microplus* Bm86 antigen in cattle vaccines. The administered silica vesicle nanovaccine accumulated at the injected site and was detected in the lymph nodes after 28 days of the inoculation in small animal model. Excitingly, this nanovaccine elicited antibody responses and did not cause any adverse effect in major vital organ tissues. This study concluded that silica vesicles should be considered as an adjuvanted delivery vehicle for the development of safe and effective veterinary subunit vaccines.

In this special issue, we also published two review articles that described advantages of novel adjuvants to improve the vaccines efficacy. Suitable adjuvants for delivering/improving vaccines efficacy are needed, especially in veterinary health, where animal vaccine adjuvants are limited. Heat shock proteins 90 kDa (Hsp90s) are highly conserved stress-responsive proteins present in all living organisms. Hsp90s have the capability to bind to surface receptors and activate cellular functions related to immune response and can act as a potent immunomodulator. Corigliano et al. comprehensively reviewed studies that used Hsp90s as an adjuvant alone, as well as complexed or fused to antigen to improve vaccine efficacy. Importantly, this review highlighted mechanisms of action wherein Hsp90s activated and modulated professional antigen-presenting cells through on interaction with CD91 and Toll-like receptors, scavenger receptor family, and antigen/peptide cross-presentation. Polyphosphazenes are high molecular weight, water-soluble, synthetic polymers that have been recently investigated as adjuvant to improve vaccines induced immune responses. Chand et al. presented the polyphosphazenes basic structure, synthesis methods, formulation preparation, and its mechanisms of action to augment vaccine efficacy. This review highlighted wherein polyphosphazenes were used as an adjuvant to improve the viral and bacterial antigens immunogenicity compared across different routes of vaccine administration. Additionally, this review article described advantages of combining polyphosphazenes with other adjuvants and the effect these adjuvant combinations had on induction of immunity. Together, these articles highlight recent advances on adjuvants in veterinary vaccines.

## Author Contributions

SR drafted and revised. HLW, KT, and JH have edited the article. All authors approved article for the publication.

## Conflict of Interest

The authors declare that the research was conducted in the absence of any commercial or financial relationships that could be construed as a potential conflict of interest.

